# Donor Heart Preservation for Heart Transplantation: Single-Center Experience with Three Different Techniques

**DOI:** 10.3390/jcm14041108

**Published:** 2025-02-09

**Authors:** Andrea Lechiancole, Gregorio Gliozzi, Sandro Sponga, Pierluigi Visentin, Antonio Beltrami, Daniela Piani, Giovanni Benedetti, Cristian Daffarra, Andriy Dralov, Matteo Meneguzzi, Giorgio Guzzi, Alessandro Di Lorenzo, Laura Stella, Uberto Bortolotti, Ugolino Livi, Igor Vendramin

**Affiliations:** 1 Cardiothoracic Department, Azienda Sanitaria Universitaria Friuli Centrale, P. le S.M. Misericordia 15, 33100 Udine, Italy; gregorio.gliozzi@asufc.sanita.fvg.it (G.G.); sandro.sponga@asufc.sanita.fvg.it (S.S.); daniela.piani@asufc.sanita.fvg.it (D.P.); giovanni.benedetti@asufc.sanita.fvg.it (G.B.); cristian.daffarra@asufc.sanita.fvg.it (C.D.); andriy.dralov@asufc.sanita.fvg.it (A.D.); matteo.meneguzzi@asufc.sanita.fvg.it (M.M.); giorgio.guzzi@asufc.sanita.fvg.it (G.G.); uberto48@gmail.com (U.B.); igor.vendramin@uniud.it (I.V.); 2 Department of Medicine, University of Udine, via Colugna 50, 33100 Udine, Italy; antonio.beltrami@uniud.it (A.B.); dilorenzoalessandro1@gmail.com (A.D.L.); laura_esse_@libero.it (L.S.); 3 Institute of Pathology, Azienda Sanitaria Universitaria Friuli Centrale, University Hospital of Udine, P. le S.M. Misericordia 15, 33100 Udine, Italy; pierluigi.visentin@asufc.sanita.fvg.it

**Keywords:** heart transplantation, donor organ preservation, cold storage, controlled hypothermia, ex situ normothermic perfusion

## Abstract

**Objectives:** In addition to traditional ice-cold storage (ICS), other techniques are emerging in the field of donor heart preservation for heart transplantation (HTx). However, in many centers, it could be difficult to justify their use, due to the higher costs and the greater technical complexity compared to ICS. This study aims to analyze the results obtained for HTx at our center employing ICS, controlled hypothermia with Paragonix SherpaPak (PSP), and ex vivo normothermic perfusion with Organ Care System (OCS) as donor graft preservation strategies. **Methods**: All HTx performed at the University Hospital of Udine, between January 2020 and August 2024, was analyzed and patient outcomes and complications after HTx were assessed. Endomyocardial biopsies were performed in donor hearts immediately after retrieval (T_0_), before implantation (T_1_), and at reperfusion (T_2_) to evaluate signs of myocardial damage. **Results**: Overall, 100 patients were transplanted with a donor heart preserved with ICS (*n* = 30), PSP (*n* = 36), or OCS (*n* = 34). Compared to ICS, PSP and OCS recipients showed a higher median IMPACT score (5 vs. 8 vs. 7, respectively, *p* = 0.05) and tended to have a higher rate of bridging to HTx with a long-term ventricular assist device (7% vs. 17% vs. 29%, *p* = 0.06). OCS was more commonly used in cases of expected ischemic time >4 h compared to ICS and PSP (*p* < 0.01). Histologically, severe degrees of cellular damage were higher in those hearts preserved with ICS. The 30-day mortality was 3% vs. 6% vs. 9% in ICS, PSP, and OCS groups, respectively (*p* = 0.65). Moderate-to-severe primary graft dysfunction was 37% vs. 11% vs. 17% (*p* = 0.03) in the three groups. **Conclusions**: PSP and OCS seem to be valid alternatives to traditional ICS, and their use could be strongly considered, particularly in the most complex and critical settings, until further data are available on more patient experiences.

## 1. Introduction

For over 50 years, heart transplantation (HTx) has been performed utilizing organs preserved with ice-cold storage (ICS) [1]. The simplicity of use and the extremely low cost are the main characteristics that enable traditional ICS to continue to be the most commonly used method of graft preservation. However, the main drawbacks of this technique are the risk of freezing damage to the donor organ, and the negative effect on function if graft ischemic time is longer than four hours [1,2,3,4,5,6,7,8,9].

In order to limit the gap between patients on being on a waiting list and having HTx performed, every effort should be made in expanding the donor pool. In fact, despite the fact that in 2023 in Italy, a total of 370 HTxs were performed, 669 patients were still on a waiting list, and 45 died waiting for HTx [10]. Interestingly, the HTx scenario is expected to become increasingly complex; in fact, the improvement of pharmacological and invasive treatments has increased the age and comorbidities of potential HTx candidates. Moreover, the allocation policy in Italy, similarly to that of other countries, prioritizes recipients in a worse clinical condition, particularly those on extra-corporeal membrane oxygenation (ECMO) and other short-term mechanical circulatory supports.

On the other hand, the employment of donors with “extended criteria” to limit donor shortages could impair outcomes after HTx. In this setting, donor heart preservation strategy could have a significant impact on HTx results.

In the last decade, various techniques have been proposed for donor heart preservation, with the aim of eliminating the limitations related to ICS [11]. Among these, the Organ Care System (OCS; TransMedics Inc., Andover, MA, USA), perfusing the donor heart with a normothermic enriched solution of oxygenated donor blood, allows both a significant reduction in graft ischemic time and continuous ex situ assessment of graft perfusion and metabolism.

Another method currently used for graft preservation is the Paragonix SherpaPak device (PSP; Paragonix Technologies, Inc., Cambridge, MA, USA) which ensures a uniform cooling of the organ at a relatively constant temperature between 4 and 8 °C, thus minimizing the risk of ice-cold temperature exposure.

Despite the number of donor hearts preserved with these new approaches increasing, both PSP and OCS are not yet considered as valuable alternatives to replace ICS, mostly because of their technical complexity or for economical and logistical reasons [11].

Since in the literature, there is a paucity of data regarding the use and results of different graft preservation techniques among HTx institutions, the aim of this study was to report the experience of our center, analyzing indications, rationale, and the results of their employment. Moreover, myocardial samples were obtained and analyzed to verify the histological variations related to the different modalities of graft preservation.

## 2. Patients and Methods

### 2.1. Patient Population

The study was approved by the Institutional Review Board (Clinical Registration Number: 317/2024) and informed consent was waived due to its retrospective nature.

In the University Hospital of Udine HTx Center of Udine, in one of the Proceed II trial recruiting Centers [12], the OCS has been used since 2007, while the PSP was introduced in 2020.

All consecutive HTx performed between January 2020 and August 2024 were included in this study. The patient population was divided into three groups according to the technique of donor graft preservation using either ICS, PSP, or OCS.

The aims of this study were to evaluate the following, according to a graft preservation strategy: 1—the baseline characteristics of donors and recipients, 2—histology data of donor hearts, and 3—clinical outcomes after HTx.

### 2.2. Definition of Terms

Extended-criteria donor characteristics were considered those derived from the OCS Heart Expand Trial [13]: (1) a total ischemic time ≥4 h or (2) a total ischemic time ≥2 h with at least one of the following: over 55 years of age, a downtime >20 min, left ventricular ejection fraction (LVEF) between 40% and 50%, left ventricular posterior wall thickness 12 to 16 mm, or coronary luminal irregularities.

Definitions for primary graft dysfunction (PGD), renal failure, and rejection episodes were those currently accepted and available from the most recent guidelines and pertinent literature [1,13,14,15]_._

Baseline donor and recipient characteristics, clinical results, and histological data of the donor graft were compared between the two groups.

### 2.3. Surgical Technique

All donor hearts were arrested with St. Thomas II solution in the aortic root, and cardiectomy was performed in a standard fashion.

### 2.4. ICS Preservation

Once retrieved, the donor heart is placed into a sterile bag filled with 1000 mL of preservation solution at 4 °C, sealed in a second bag containing 1000 mL of cold solution, and then placed in a third bag. Then, the heart is placed in a rigid sterile container full of cold saline inserted into a cooler filled with ice slush for transportation. One surgeon and a nurse are generally sufficient to perform this procedure.

### 2.5. PSP Preservation

PSP consists of an internal and an external canister. The donor heart is placed into the internal canister, which is filled with St. Thomas II cardioplegic solution, at a temperature between 4 and 8 °C, and then placed into the external canister, creating an insulating air chamber, surrounded by disposable cooling ice packs that freeze at a temperature higher than 0 °C. A thermometer connected with the internal canister allows for a continuous monitoring of the solution temperature. PSP preservation requires two surgeons and a nurse.

### 2.6. OCS Preservation

The donor heart, continuously perfused by an oxygenated, enriched donor blood solution, is maintained at 34 °C and beating. The viability of the graft is assessed through the detection of mean aortic pressure, coronary flow, and the total arterial and arterio-venous ratio lactate profile. An arterial lactate level > 5 mmol/L is generally accepted as a valuable parameter for discarding the graft; likewise, an unfavorable arterio-venous lactate ratio, as evidenced by a higher venous than arteriosus lactate concentration, indicates myocardial suboptimal perfusion. OCS preservation requires a team composed of a trained surgeon, an assistant surgeon, a trained perfusionist, and a nurse.

All HTxs were performed using the bicaval anastomosis technique. Postoperative immunosuppression was based on previously described protocols [16].

### 2.7. Rationale for Organ Preservation Strategy

**Figure 1:**
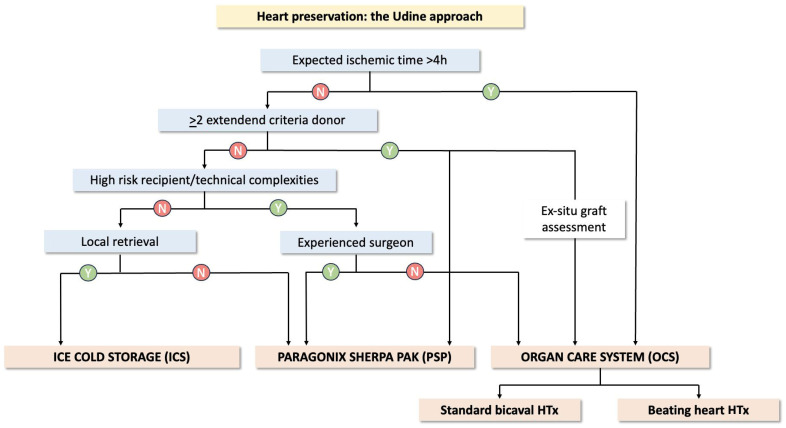
Algorithm of preservation strategies. Y = yes and N = No.

As reported in Figure 1, in our institution, three main factors influenced the preservation strategy: 1—expected graft ischemic time, 2—the number of extended criteria for donors, 3—the technical complexity of the HTx.

As a general rule, PSP and OCS were preferred over ICS when the number of extended donor criteria was more than 1, and in technically complex HTx, with OCS more commonly adopted in cases of expected ischemic time >4 h.

### 2.8. Tissue Sampling

Based on our institutional protocol, and with the informed consent of HTx candidates, epi-myocardial biopsies (2.0 to 5.0 mm) were routinely taken in donor grafts from the left ventricular wall at the apex using a 16 g bioptome (Bard Peripheral Vascular Inc. Temple, AZ, USA) at time of procurement (T_0_), just prior to implantation (T_1_) and immediately after the release of the aortic clamp (T_2_) in all groups. Analysis of the biopsy samples was performed by pathologists unaware of the technique of donor graft preservation.

### 2.9. Histological Analysis

For histology, myocardial samples were fixed in 10% buffered formalin, embedded in paraffin, and cut 5 µm thick. Sections of myocardial biopsies were stained in haematoxylin and eosin and analyzed to assess the presence and degree of endothelial swelling, edema, and myocyte injury. To quantify myocardial tissue damage, a specific histological score was used: scores 0, 1, and 2 corresponded to an absence of lesions, mild lesions (≤20%), and severe lesions (>20%), respectively [17].

### 2.10. Statistical Analysis

The population of the study was divided into three groups according to the donor heart preservation strategy. Continuous variables are expressed as the mean ± standard deviation (SD) or median and interquartile range (IQR), according to the data distribution, and analyzed using the Shapiro–Wilk test to verify the normal distribution. Categorical variables are presented as absolute numbers and percentages. The Kruskal–Wallis test was used to compare continuous variables between groups. Comparison of categorical variables was performed by Fisher’s exact test. A *p* value less than 0.05 was considered significant.

All statistical analyses were performed using the Statistical Package for Social Sciences (SPSS) program (Chicago, IL, USA).

## 3. Results

### 3.1. Baseline Patient Characteristics

A total of 100 patients underwent HTx in our institution during the study period. HTx was performed using donor grafts preserved with ICS (*n* = 30), PSP (*n* = 36), and OCS (*n* = 34), respectively. Baseline clinical characteristics of recipients and donors are reported in detail in Table 1.

ICS, PSP, and OCS recipient groups differed for median age (56 vs. 58 vs. 64 years, *p* = 0.005) and median Index for Mortality Prediction After Cardiac Transplantation (IMPACT) score (5 vs. 8 vs. 7, *p* = 0.05). Compared to ICS, PSP and OCS groups tended to have higher rates of bridging with a durable ventricular assist device (VAD) (7% vs. 17% vs. 29%, *p* = 0.06).

Median donor age (52, 51, and 56 years, *p* = 0.43) and the rate of extended-criteria donor employment (60% vs. 61% vs. 71%, *p* = 0.61) were similar between ICS, PSP, and OCS groups, respectively. Grafts from donors with ≥2 extended criteria tended to be more likely preserved with PSP (17%) and OCS (21%) than ICS (10%) (*p* = 0.51).

As shown in Table 2, compared to the ICS and PSP groups, the OCS group showed a significantly higher rate of expected graft ischemic time >4 h (17% vs. 28% vs. 76%, *p* < 0.01) and longer median distance of donor retrieval site from our center (117 km vs. 300 km vs. 493 km, *p* < 0.01). However, higher rates of ischemic time >4 h were more frequent in the PSP group (44%), than the ICS (23%) and OCS (0%) groups, respectively (*p* < 0.01).

### 3.2. Histological Analysis

Biopsies were performed in 66 donor hearts: 21 from the ICS, 22 from the PSP, and 23 from the OCS group, respectively.

**Figure 2:**
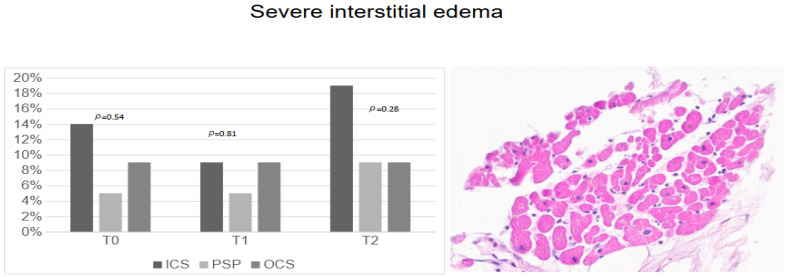
Severe interstitial edema according to preservation techniques.

Severe interstitial edema rates were almost similar at T_0_, T_1,_ and T_2_ in the PSP and OCS groups (Figure 2), while hearts preserved with ICS tended to be more commonly affected by severe interstitial edema at T_0_ and T_2_ (*p* = 0.54 and *p* = 0.28).

**Figure 3:**
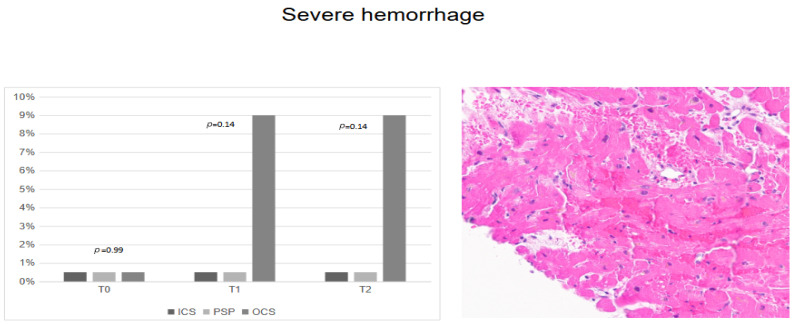
Severe hemorrhages according to preservation techniques.

Severe hemorrhages (Figure 3) were more likely to be detected in OCS groups at T_1_ and T_2_, with no statistical significance (*p* = 0.14).

**Figure 4:**
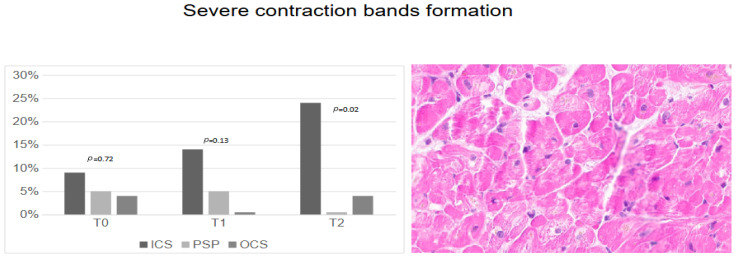
Severe contraction band formation according to preservation techniques.

At a cellular level, ICS showed an increasing rate of severe contraction band formation (Figure 4), which became significantly higher at T_2_ (24%) compared to for PSP (0%) and OCS (4%), *p* = 0.02.

**Figure 5:**
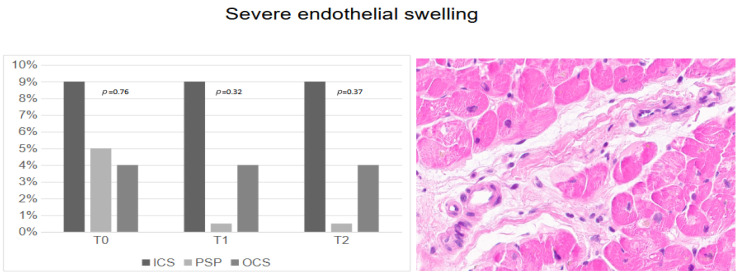
Severe endothelial swelling according to preservation techniques.

The rates of endothelial swelling (Figure 5) were not statistically different at T_0_, T_1,_ and T_2_ for all groups.

### 3.3. Clinical Outcomes

Perioperative results are reported in Table 3. After HTx, 30-day mortality was 3% (*n* = 1) for multiorgan failure (MOF) in the ICS group, 6% (*n* = 2) for MOF and PGD in the PSP group, and 9% (*n* = 3) for MOF in two cases and PGD in one in the OCS group, respectively (*p* = 0.64).

Among the ICS, PSP, and OCS groups, the cumulative incidence of right ventricle and moderate-to-severe left ventricle PGD was 37% vs. 11% vs. 17% (*p* = 0.03).

Post-HTx veno-arterial extracorporeal membrane oxygenation (ECMO) was employed in two patients of the ICS group (because of severe PGD), in three patients of the PSP group (two for severe PGD and one for sepsis) and in four patients of the OCS group (because of severe PGD in three cases and of severe vasoplegia in one). In all cases, a peripheral femoro-femoral cannulation was used.

Early (up to 72 h after HTx) atrial fibrillation episodes were detected in 47% vs. 19% vs. 23% of patients of the ICS, PSP, and OCS groups (*p* = 0.09), while early complete atrioventricular block was reported in four ICS and two PSP patients, and one OCS patient, and all but one ICS patient required definite pacemaker implantation.

At 1 year, overall mortality was 10% vs. 8% vs. 20% for the ICS, PSP, and OCS groups, respectively (*p* = 0.26). After 30 days, causes of death were sepsis (*n* = 1) in the ICS group, cerebrovascular accident (*n* = 1) in the PSP group, and sepsis (*n* = 2), cerebrovascular accident (*n* = 1), and MOF (*n* = 1) in the OCS group, respectively.

Grade ≥ 2 rejection episodes within 1 year after HTx were observed in 10 patients (33.3%) of the ICS vs. 10 (28%) of PSP vs. 8 (24%) of the OCS group (*p* = 0.68, respectively). All of them required hospital readmission and were treated with high-dose steroid pulse therapy.

The 1-year incidence of grade ≥ 2 rejection was 50% among the 24 patients who received a graft with severe histological deteriorations, and 21% among the others (*p* = 0.006).

## 4. Discussion

This study evaluates the results of HTx using ICS, PSP, and OCS, three different techniques of donor heart preservation, available at our center. Compared to ICS, PSP and OCS were more commonly used in more technically challenging situations, such as in those with previous cardiac operations or bridged with a long-term VAD, and in more clinically compromised HTx candidates. The OCS was extensively employed when expected ischemic time was > 4 h, since longer ischemic times have a negative impact on the development of PGD, particularly in grafts from donors selected with extended criteria.

Comparing these three preservation strategies, we have observed similar early results with no differences in 30-day mortality, despite the different settings of their employment. However, in PSP and OCS patients, a trend to lower rates of moderate-to-severe PGD was observed. In fact, histological findings confirmed the superiority of PSP and OCS in better preserving the donor heart, as indicated by the extremely low finding of severe damage in both myocardial and endothelial cells in grafts stored with these techniques. Moreover, the near-absence of severe myocardial contraction band formation after reperfusion in PSP and OCS transplanted grafts appears to indicate that donor hearts preserved under controlled hypothermia and ex situ normothermic perfusion display a superior resistance to ischemia–reperfusion damage, compared to those just stored in ice. Most likely for the same reason, recipients receiving PSP- and OCS-preserved grafts experienced a significantly lower incidence of atrial fibrillation within 72 h of HTx compared to those with ICS-preserved grafts, probably because of limited damage to the conduction system.

Despite not reaching statistical significance, patients of the OCS group had a higher 1-year mortality. This result is more likely due, compared to the poor quality of the donor graft, to the fact that in this group, the recipient population was older and more fragile, as also suggested by the causes of death.

In this series, severe histological alterations seem to confer a higher risk of developing a rejection grade ≥2 during the first year after HTx. Although analysis of the limited amount of data fails to reach statistical significance, our results seem to suggest that tissue damage could amplify the immune response of the recipient towards the donor graft, promoting a local inflammatory reaction that increases the exposure of donor antigens, as previously suggested [18]. The correlation between histological alterations and development of coronary artery vasculopathy and of late graft dysfunction could be an intriguing area of investigation, but in our series, this is limited by the short follow-up period.

Interestingly, the tendency towards higher rates of severe interstitial edema and endothelial swelling at T_0_ in grafts preserved with ICS could be related to a larger volume dose of cardioplegia solution during donor heart procurement (up to 2.0 L), instead of a standard dose of 1 L in PSP and 0.5 to 1 L in OCS grafts.

The results of this study further confirm the data reported from larger series. Data from Guardian Registry analyses [19,20] indicate that PSP preservation has proven to be more effective than ICS in reducing the rate of PGD and need for mechanical circulatory support after HTx, although it did not affect 1-year survival. Interestingly, a recent study demonstrated a better cost-effectiveness of PSP over ICS [21]. In fact, by reducing the incidence of severe PGD (5.7% vs. 16.1%, *p* = 0.03) and the need for circulatory support after HTx (21.8% vs. 40.2%, *p* = 0.009), PSP preservation contributed to limit the duration of recipient hospital stay and consequent costs [21].

It is also worth underlining that with regard to PSP, the favorable results reported in this study are obtained despite grafts having an ischemia time >4 h in 44% of cases. This figure further supports recent studies that demonstrate better results when donor hearts are preserved with PSP with ischemic times >4 h, compared to ICS [22]. By avoiding any risk of freezing damage, showing a tendency towards better graft protection, and allowing rapid recovery from ischemia–reperfusion injury [23,24], PSP could safely be employed in complex situations such as in patients supported by durable a VAD [22], for whom ischemic time could be prolonged for unexpected technical issues.

The non-inferiority of ex situ normothermic perfusion compared to ICS has been shown in the PROCEED II controlled trial, where the OCS was demonstrated to significantly reduce graft ischemic time [12]. Moreover, four hearts of the OCS group were discarded after metabolic assessment during perfusion, and histological analysis revealed signs of infarction, contusion, and severe unrecognized left ventricular hypertrophy [12]. The EXPAND trial [13], evaluating the impact of OCS preservation in donor hearts selected using extended criteria, found that out of 173 such grafts, 150 (87%) were utilized for HTx. The 30-day post-HTx survival rate was 97%, and the incidence of severe PGD was 6.7%.

A previous study from our group demonstrated that OCS, compared to ICS in preserving grafts from extended criteria donors, seemed to guarantee better hemodynamic status and lower rates of complications after HTx, allowing a 5-year survival of 100% vs. 73, respectively (*p* = 0.04) [25].

Although a randomized controlled trial is lacking, the effectiveness of PSP over ICS in heart preservation is supported by robust data of the Guardian Registry; furthermore, according to the ISHLT Consensus Statement on thoracic organ procurement [8], in which the maintenance of a storage temperature between 5 and 10 °C is recommended to avoid potential ice-related injuries to graft tissues, PSP is acquiring a promising role, as compared to ICS. On the other hand, OCS proved to dramatically reduce graft ischemic times, allowing for an increase in acceptance rates of extended-criteria donor organs. Moreover, OCS favors a stress-free and meticulous preparation of the recipients, even in technically complex re-HTx, while the donor graft is maintained as perfused. However, despite PSP and OCS being reported as safe and effective preservation techniques, a unanimous consensus on the indications for their use is still lacking. Indeed, reluctancy to their more extended use most likely derives from hesitation to deviate from a consolidated clinical practice, higher costs, and the need to acquire adequate technical expertise with these new methods. Based on these considerations, a possible solution could be represented by a careful use of each technique to provide a fair cost–benefit ratio.

In order to optimize technical and economic resources, we have developed an algorithm which could facilitate the choice of the most appropriate graft preservation method. As graphically reported in Figure 1, the variables that mainly influenced the choice of the preservation technique were as follows: expected ischemic time >4 h, extended criteria for donors, and recipient characteristics. In our experience, grafts were more commonly preserved with ICS in low-risk donors and recipients, compared to PSP and OCS. The ex situ normothermic perfusion, however, was preferred over static cold storage techniques when expected ischemic times were longer than 4 h, and when HTx was considered particularly challenging, to facilitate careful chest re-entry and recipient cardiectomy. In our opinion, this aspect is of paramount importance in facilitating in-training implanting surgeons. Based on these results, PSP has recently become the standard cold storage technique, with ICS almost only employed in on-site donor heart retrieval.

This study has some limitations which must be acknowledged. It is a retrospective study and therefore lacks the potential relevance of a prospective, randomized analysis. Moreover, the comparison of the effectiveness of different methods of donor graft preservation was performed in different clinical scenarios. This was deliberately chosen as the rationale of the study and reflects our center’s policy regarding the employment of traditional ICS and more recent and modern preservation devices. This study also has the limitation of the relatively small number of patients enrolled, which precluded sophisticated statistical analyses, but it is in further progress and the present data are to be considered as a preliminary contribution to an important and not yet completely clarified issue.

Another limitation that may be considered is the limited number of grafts from which epi-myocardial biopsy samples were obtained with possible sampling errors. However, during the procedure, a well-standardized technique was adopted by experienced operators, and both the number and size of tissue samples were adequate for a reliable histological analysis.

In conclusion, our results indicate that PSP and OCS used in the preservation of donor hearts seem to be valid alternatives to traditional ICS and their use can be considered to ensure an excellent relationship between clinical need and effectiveness. Furthermore, OCS and PSP efficacy appears to also be supported by the histological findings observed in this study in serial epi-myocardial biopsy samples.

We believe that our preliminary observations may provide promising perspectives in terms of the rationale of use of modern preservation methods, which have to be either validated or challenged by similar studies on larger patient subsets.

## Figures and Tables

**Table 1:** Baseline recipients’ characteristics.

	ICS (*n* = 30)	PSP (*n* = 36)	OCS (*n* = 34)	*p*
Age—years, median (IQR)	56 (49–62)	58 (44–62)	64 (56–67)	0.005
Male sex, *n* (%)	21 (70)	26 (72)	27 (79)	0.66
Diabetes mellitus, *n* (%)	3 (10)	5 (14)	9 (26.5)	0.18
Dyslipidemia, *n* (%)	10 (33)	10 (28)	16 (47)	0.23
COPD, *n* (%)	3 (10)	5 (6)	3 (9)	0.78
≥moderate CKD, *n* (%)	5 (17)	4 (11)	6 (18)	0.71
Dialysis, *n* (%)	2 (7)	2 (6)	1 (3)	0.78
sPAP—mmHg, median (IQR)	42 (30–49)	36 (27–45)	34 (27–39)	0.10
Etiology				0.15
*Idiopathic dilative, n (%)*	*8 (27)*	*8 (22)*	*13 (38)*	
*Ischemic, n (%)*	*9 (30)*	*11 (31)*	*10 (29)*	
*Valvular, n (%)*	*3 (10)*	*2 (6)*	*2 (6)*	
*Restrictive, n (%)*	*3 (10)*	*7 (19)*	*1 (3)*	
*Other, n (%)*	*7 (23)*	*8 (22)*	*8 (24)*	
Previous cardiac surgery, *n* (%)	13 (43)	15 (42)	22 (65)	0.11
L-VAD, *n* (%)	2 (7)	6 (17)	10 (29)	0.06
Re-HTx, *n* (%)	2 (7)	3 (8)	5 (15)	0.52
ECLS, *n* (%)	4 (13)	10 (28)	5 (15)	0.24
IABP, *n* (%)	4 (13)	6 (17)	5 (15)	0.93
IMPACT score, median (IQR)	5 (2–8)	8 (4–13)	7 (5–12)	0.05

IQR = interquartile range; COPD = chronic obstructive pulmonary disease; CKD = chronic kidney disease; sPAP = systolic pulmonary artery pressure; Lt VAD = long-term ventricular assist device; Re-HTx = heart re-transplantation; ECLS = extracorporeal life support; IABP = intra-aortic balloon pump; IMPACT = Index for Mortality Prediction After Cardiac Transplantation.

**Table 2:** Donors and procedural data.

	ICS (*n* = 30)	PSP (*n* = 36)	OCS (*n* = 34)	*p*
Age—years, median (IQR)	52 (45–57)	51 (44–58)	56 (49–59)	0.43
Age > 55 years, *n* (%)	12 (40)	14 (39)	18 (53)	0.43
Male sex, *n* (%)	14 (47)	14 (39)	13 (38)	0.75
Cause of death				0.11
*Cerebrovascular accident, n (%)*	*12 (40)*	*26 (72)*	*26 (76)*	
*Trauma, n (%)*	*12 (40)*	*5 (14)*	*6 (18)*	
*Anoxia, n (%)*	*2 (7)*	*4 (11)*	*2 (6)*	
*Other, n (%)*	*2 (13)*	*1 (3)*	*0 (0)*	
Gender mismatch, *n* (%)	10 (33)	8 (22)	8 (23)	0.48
Extended-criteria donor, *n* (%)	18 (60)	22 (61)	24 (71)	0.61
≥2 extended criteria, *n* (%)	3 (10)	6 (17)	7 (21)	0.51
Downtime ≥ 20 min, *n* (%)	5 (17)	6 (17)	4 (12)	0.82
LVEF ≥ 40% and ≤50%, *n* (%)	0 (0)	1 (3)	2 (6)	0.38
LVPP > 12 mm, *n* (%)	2 (7)	1 (3)	4 (12)	0.34
CAD, *n* (%)	1 (3)	5 (14)	4 (12)	0.32
Diabetes mellitus, *n* (%)	1 (3)	0 (0)	0 (0)	0.31
Drug abuse, *n* (%)	1 (3)	2 (6)	1 (3)	0.79
Procedural				
Expected IT > 4 h, *n* (%)	5 (17)	10 (28)	26 (76)	<0.01
Actual IT > 4 h, *n* (%)	7 (23)	16 (44)	0 (0)	<0.01
IT—min, median (IQR)	210 (156–253)	229 (172–251)	138 (109–150)	<0.01
Distance from retrieval site—Km, median (IQR)	117 (0–351)	300 (115–433)	493 (379–874)	<0.01
CPB time—min, median (IQR)	202 (163–228)	197 (154–221)	206 (180–239)	0.71

IQR = interquartile range; LVEF = left ventricular ejection fraction; LVPW = left ventricular posterior wall thickness; CAD = coronary artery disease; IT = ischemic time; Km = Kilometers; CPB = cardiopulmonary bypass.

**Table 3:** Outcomes.

	ICS (*n* = 30)	PSP (*n* = 36)	OCS (*n* = 34)	*p*
30-day mortality, *n* (%)	1 (3)	2 (6)	3 (9)	0.65
≥moderate LV and RV PGD, *n* (%)	11 (37)	4 (11)	6 (17)	0.03
*Moderate LV PGD, n (%)*	*7 (23)*	*2 (6)*	*1 (3)*	
*Severe LV PGD, n (%)*	*2 (7)*	*2 (6)*	*3 (9)*	
*RV PGD, n (%)*	*2 (7)*	*0 (0)*	*2 (6)*	
ECLS support, *n* (%)	2 (7)	3 (8)	4 (12)	0.76
Complete AV block, *n* (%)	3 (10)	2 (6)	1 (3)	0.51
AF within 72 h, *n* (%)	14 (47)	7 (19)	8 (23)	0.09
MV—hours, median (IQR)	48 (24–72)	37 (24–72)	48 (24–140)	0.45
RRT, *n* (%)	6 (20)	9 (25)	11 (32)	0.52
Re-exploration for bleeding, *n* (%)	5 (17)	13 (36)	7 (21)	0.16
Grade ≥ rejection episodes, *n* (%)	10 (33)	10 (28)	8 (24)	0.68
1-year mortality, *n* (%)	3 (10)	3 (8)	7 (20)	0.26

LV = left ventricle; RV = right ventricle; PGD = primary graft dysfunction; ECLS = extracorporeal life support; AV = atrio-ventricular; MV = mechanical ventilation; IQR = interquartile range; RRT = renal replacement treatment.

## Data Availability

The raw data supporting the conclusions of this article will be made available by the authors on request.

## Abbreviations

HTx	Heart Transplantation
PDG	Primary Graft Dysfunction
MCS	Mechanical Circulatory Support
ICS	Ice-Cold Storage
PSP	Paragonix SherpaPak
OCS	Organ Care System
